# Adverse Outcomes after Major Surgeries in Patients with Diabetes: A Multicenter Matched Study

**DOI:** 10.3390/jcm8010100

**Published:** 2019-01-16

**Authors:** Chao-Shun Lin, Chuen-Chau Chang, Yuan-Wen Lee, Chih-Chung Liu, Chun-Chieh Yeh, Yi-Cheng Chang, Ming-Tsang Chuang, Tzu-Hao Chang, Ta-Liang Chen, Chien-Chang Liao

**Affiliations:** 1 Department of Anesthesiology, School of Medicine, College of Medicine, Taipei Medical University, Taipei 110, Taiwan; lin.soon@gmail.com (C.-S.L.); nekota@tmu.edu.tw (C.-C.C.); yuarn438@yahoo.com.tw (Y.-W.L.); aneslcc@gmail.com (C.-C.L.); tlc@tmu.edu.tw (T.-L.C.); 2 Department of Anesthesiology, Taipei Medical University Hospital, Taipei 110, Taiwan; 3 Anesthesiology and Health Policy Research Center, Taipei Medical University Hospital, Taipei 110, Taiwan; 4 Department of Surgery, China Medical University Hospital, Taichung 404, Taiwan; b8202034@gmail.com; 5 Department of Surgery, University of Illinois, Chicago, IL 60637, USA; 6 Department of Internal Medicine, National Taiwan University Hospital, Taipei 100, Taiwan; b83401040@yahoo.com.tw; 7 Office of Information Technology, Taipei Medical University, Taipei 110, Taiwan; mtchuang@tmu.edu.tw (M.-T.C.); kevinchang@tmu.edu.tw (T.-H.C.); 8 Graduate Institute of Biomedical Informatics, Taipei Medical University, Taipei 110, Taiwan; 9 Clinical Big Data Research Center, Taipei Medical University Hospital, Taipei 110, Taiwan; 10 School of Chinese Medicine, College of Chinese Medicine, China Medical University, Taichung 404, Taiwan; 11 Department of Anesthesiology, Shuan Ho Hospital, Taipei Medical University, New Taipei City 235, Taiwan

**Keywords:** adverse outcomes, diabetes, fasting glucose, surgeries

## Abstract

The impact of diabetes on perioperative outcomes remains incompletely understood. Our purpose is to evaluate post-operative complications and mortality in patients with diabetes. Using the institutional and clinical databases of three university hospitals from 2009–2015, we conducted a matched study of 16,539 diabetes patients, aged >20 years, who underwent major surgery. Using a propensity score matching procedure, 16,539 surgical patients without diabetes who underwent surgery were also selected. Logistic regressions were used to calculate the odds ratios (ORs) with 95% confidence intervals (CIs) for post-operative complications and in-hospital mortality associated with diabetes. Patients with diabetes had a higher risk of postoperative septicemia (OR 1.33, 95% CI 1.01–1.74), necrotizing fasciitis (OR 3.98, 95% CI 1.12–14.2), cellulitis (OR 2.10, 95% CI 1.46–3.03), acute pyelonephritis (OR 1.86, 95% CI 1.01–3.41), infectious arthritis (OR 3.89, 95% CI 1.19–12.7), and in-hospital mortality (OR 1.51, 95% CI 1.07–2.13) compared to people without diabetes. Previous admission for diabetes (OR 2.33, 95% CI 1.85–2.93), HbA1c >8% (OR 1.96, 95% CI 1.64–2.33) and fasting glucose >180 mg/dL (OR 1.90, 95% CI 1.68–2.16) were predictors for post-operative adverse events. Diabetes patients who underwent surgery had higher risks of infectious complications and in-hospital mortality compared with patients without diabetes who underwent similar major surgeries.

## 1. Introduction

Due to the increasing number of diabetes patients, their consumption of medical resources and economic burden has attracted attention all over the world [1,2]. Medical complications from diabetes have been reduced thanks to various interventions, including education, early screening, health examinations, and medications [3,4]. However, perioperative complications after cardiac and non-cardiac surgeries in patients with diabetes remain a clinical problem, even though surgical procedures have progressed [5,6].

The impact of diabetes history, elevated hemoglobin A1c (HbA1c), and higher fasting glucose on clinical outcomes after major surgeries has been evaluated in many studies, which have shown increased risks of perioperative mortality and complications including infection, stroke, and death in patients with diabetes compared to those without diabetes [7,8,9,10,11,12,13]. Conversely, some studies have indicated that diabetes mellitus or pre-operative glucose concentration did not relate to perioperative morbidity or mortality [14,15,16]. A recent review did not find a significant difference in perioperative outcomes in people with hyperglycemia, but did find post-operative adverse events in people who experienced hypoglycemia [17]. However, there are several limitations in the above quantitative studies, such as inadequate control for confounding factors [9,10,11,14,16], focusing on specific surgical procedures [7,8,9,10,13,15,16], or small sample sizes of diabetes patients [10,11]. In addition, limited information was available on the perioperative outcome in patients with undiagnosed diabetes, and the association between diabetes and post-operative outcomes was not fully elucidated.

Using hospital-based medical records, we conducted this multicenter matched study to investigate the risk of complications and mortality after major surgeries in patients with and without diabetes. The impact of the glycemic status on post-operative adverse outcomes was also evaluated.

## 2. Methods

### 2.1. Source of Data

Data used in this study were obtained from the institutional and clinical database of Taipei Medical University, which contains electronic health records of more than 3 million patients from three affiliated hospitals: Taipei Medical University Hospital, Wan Fang Hospital, and Shuang Ho Hospital. Patients’ clinical information, including physicians’ diagnosis, prescriptions, physical and biochemical examinations, and medical expenditures of outpatient care, emergency care, and hospitalization have been collected since 1997. All hospitals in Taiwan are under coverage of National Health Insurance, and all medical records are reviewed by the Agency of National Health Insurance. This rigorous regulation makes the electronic health records used in this study suitable for testing our hypothesis. To protect personal privacy, all identifications of patients and physicians were scrambled. This study was evaluated and approved by the Institutional Review Board of Taipei Medical University (TMU-JIRB-201701050; TMU-JIRB-201801059).

### 2.2. Study Design

Among the 109,274 patients who underwent major inpatient surgeries (defined as procedures requiring general, epidural, or spinal anesthesia as well as hospitalization for >1 day) in 2009–2015, we examined the medical records and identified 87,710 surgical patients aged ≥20 years. Of these, 17,660 surgical patients had diabetes in the year before the index surgery.

Each patient with diabetes who underwent surgery was randomly matched to a patient without diabetes who underwent surgery, using a propensity score matched-pair procedure to adjust for age, sex, low-income status, hospital volume, coexisting medical conditions, emergency of operation, duration of surgery, type of surgery, and type of anesthesia. After propensity score matching (case-control ratio, 1:1), there were 16,539 patients with and 16,539 without preoperative diabetes.

### 2.3. Criteria and Definition

In this study, diabetes patients were defined as having at least one of the following criteria: (i) physician diagnosis of diabetes ≥2 times within 2 years of the index surgery; (ii) glycated HbA1c ≥ 6.5% within 90 days of the index surgery; (iii) fasting plasma glucose ≥ 126 mg/dL within 90 days of the index surgery. The group without diabetes was defined as people without any physician’s diagnosis of diabetes within 2 years of the index surgery, and with no medical records of HbA1c ≥ 6% or fasting glucose ≥ 110 mg/dL within 90 days of the index surgery. Diabetes-related complications and type 1 diabetes were defined as subjects with diagnostic records within 2 years of the index operation. In the further analysis, undiagnosed diabetes was defined as people without a physician’s diagnosis of diabetes, but a HbA1c ≥ 6.5% or fasting glucose ≥ 126 mg/dL within 90 days of the index operation.

According to the International Classification of Diseases, Ninth Revision, Clinical Modification (ICD-9-CM), the diagnosis codes of disease history (within 2 years of index surgery) were defined, including diabetes (ICD-9-CM 250), hypertension (ICD-9-CM 401–405), ischemic heart disease (ICD-9-CM: 410–414), cardiac dysrhythmias (ICD-9-CM 427), mental disorder (ICD-9-CM 290–319), heart failure (ICD-9-CM 428), cerebrovascular disease (ICD-9-CM 430–438), chronic obstructive pulmonary disease (ICD-9-CM 491, 492, 494), asthma (ICD-9-CM 493), and renal failure (ICD-9-CM 585). Renal dialysis was defined by administration code (D8, D9). Post-operative complications were also identified as acute myocardial infarction (ICD-9-CM 410), stroke (ICD-9-CM 430–437), acute kidney injury (ICD-9-CM 584), post-operative bleeding (ICD-9-CM 998.11), pulmonary embolism (ICD-9-CM 415), deep vein thrombosis (ICD-9-CM 451.11, 451.19, 451.2, 451.81, 451.9, 453.40–453.42, 453.8, 453.9), pneumonia (ICD-9-CM 480–486), septicemia (ICD-9-CM 038), urinary tract infection (ICD-9-CM 599.0), surgical site infection (ICD-9-CM 998.5, 996.6, 998.1, 998.3), fungal infection (ICD-9-CM 110–118), necrotizing fasciitis (ICD-9-CM 728.86), cellulitis (ICD-9-CM 682, 681, 614.3, 614.4, 528.3, 478.21, 478.71, 376.01), acute pyelonephritis (ICD-9-CM 590.1), infectious arthritis (ICD-9-CM 711.0, 711.4, 711.5, 711.6, 711.9), and osteomyelitis (ICD-9-CM 730). Length of hospital stay and medical expenditure of index surgical admission were also considered as study outcomes.

### 2.4. Statistical Analysis

**Figure A1:**
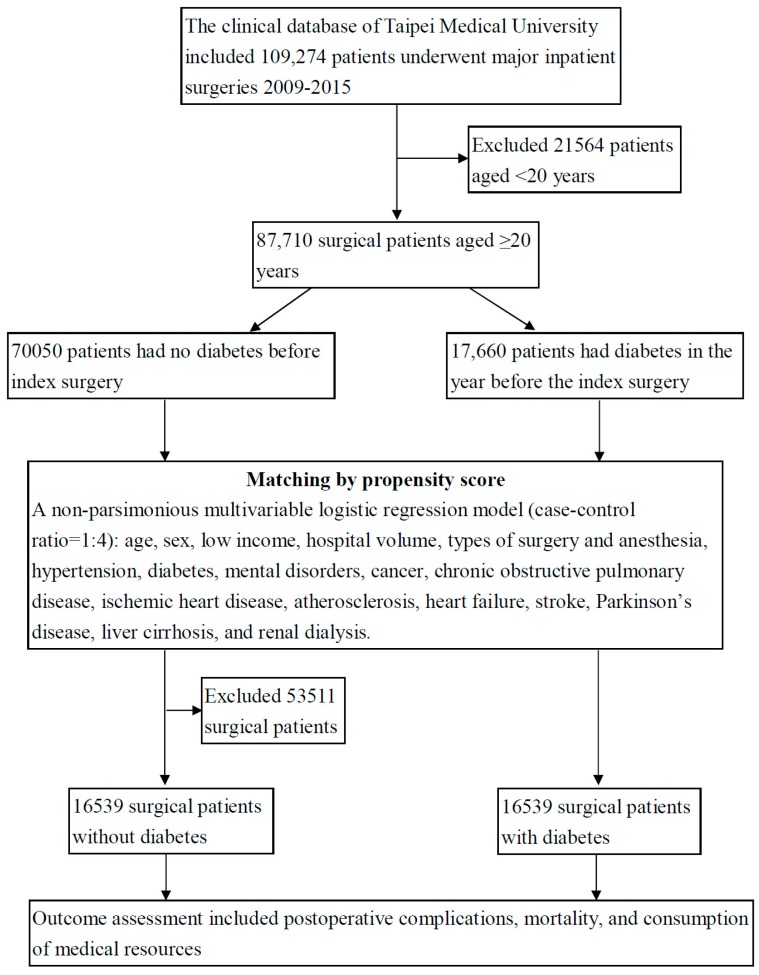
The flowchart of matching by propensity score for patients with diabetes and people without diabetes.

We used propensity score-matched pair analysis to determine the associations between pre-operative diabetes and the outcomes after surgery (Figure A1). A non-parsimonious multivariable logistic regression model was used to estimate a propensity score for surgical patients with or without diabetes. Clinical significance guided the initial choice of covariates in this model to include age, sex, low income, hospital volume, types of surgery and anesthesia, hypertension, diabetes, mental disorders, cancer, chronic obstructive pulmonary disease, ischemic heart disease, atherosclerosis, heart failure, stroke, Parkinson’s disease, liver cirrhosis, and renal dialysis. We matched diabetes patients to patients without diabetes, using a greedy matching algorithm (without replacement) with a caliper width of 0.2 SDs of the log odds of the estimated propensity score. By calculating the standardized difference of the mean or proportion for each covariate after matching, baseline covariates with a standardized difference of less than 10% were indicative of good balance between the patients with and without diabetes. Propensity score methods were used to account for selection bias when using observational data [18].

Categorical variables were summarized using frequencies (percentages), and were compared between patients with diabetes and patients without diabetes using chi-square tests. Continuous variables were summarized using the means ± standard deviations, and were compared using *t* tests. Logistic regressions were used to calculate the adjusted odds ratios (ORs) and 95% confidence intervals (CIs) of the post-operative outcomes associated with preoperative diabetes. Additional analyses stratified by age, sex, number of medical conditions, and surgery type were also performed to examine the association between diabetes and post-operative adverse events within these strata. All data management and analyses were performed using SAS Enterprise Guide software version 7.11 (SAS Institute, Cary, NC, USA); *p* values < 0.05 were considered statistically significant.

## 3. Results

**Figure A2:**
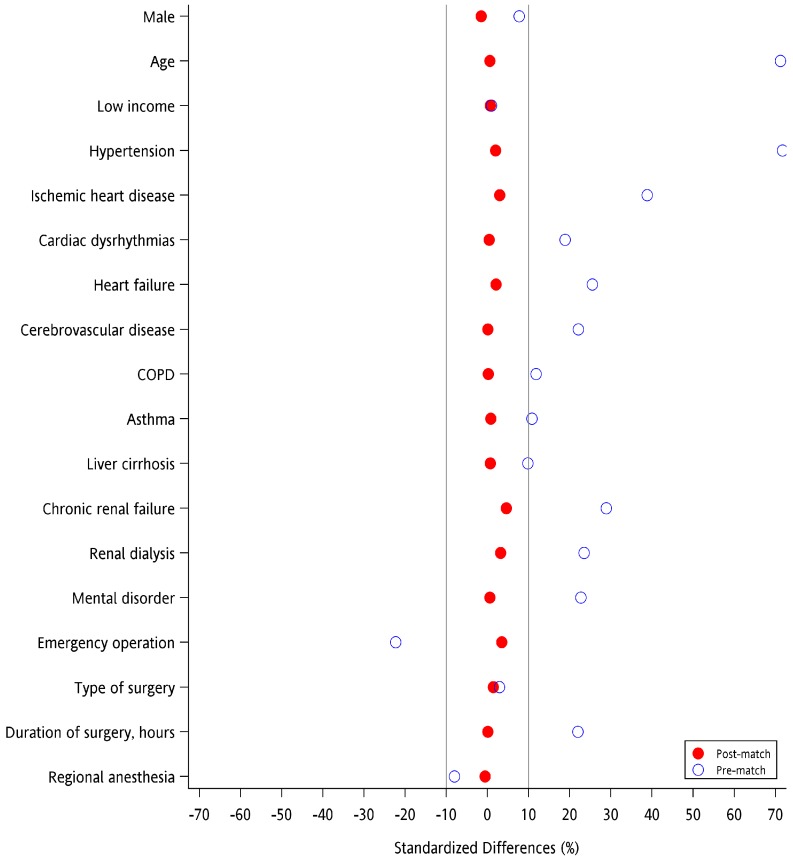
The standardized differences of baseline covariates between surgical patients with and without diabetes.

The baseline characteristics of the surgical patients with and without diabetes are shown in Table 1. After propensity-score matching, the reduced standardized differences were below 10% for baseline covariates such as age, sex, low-income status, types of surgery and anesthesia, hypertension, ischemic heart disease, cardiac dysrhythmias, mental disorders, heart failure, cerebrovascular disease, chronic obstructive pulmonary disease, asthma, and renal failure or renal dialysis between surgical patients with and without diabetes (Table 2 and Figure A2).

Compared with the control group (Table 3), patients with diabetes had a higher risk of post-operative septicemia (OR 1.33, 95% CI 1.01–1.74), necrotizing fasciitis (OR 3.98, 95% CI 1.12–14.2), cellulitis (OR 2.10, 95% CI 1.46–3.03), acute pyelonephritis (OR 1.86, 95% CI 1.01–3.41), infectious arthritis (OR 3.89, 95% CI 1.19–12.7), intensive care unit admission (OR 1.24, 95% CI 1.14–1.34), and 30 day in-hospital mortality (OR 1.51, 95% CI 1.07–2.13). Diabetes was also associated with postoperative adverse outcomes in the analyses without matching by propensity score (Table S1) and the analyses with frequency matching (Table S2).

Longer lengths of hospital stay (9.1 ± 14.5 vs. 7.1 ± 10.1 days, *p* < 0.0001) and higher medical expenditure (5167 ± 5720 vs. 4221 ± 4693 USD, *p* < 0.0001) were also noted in patients with diabetes than in patients without diabetes.

Compared with non-diabetes controls (Table 4), people with undiagnosed diabetes (OR 1.40, 95% CI 1.26–1.56), diabetes-related complications (OR 1.68, 95% CI 1.45–1.94), type 2 diabetes (OR 1.39, 95% CI 1.25–1.55), diabetes admission (OR 2.33, 95% CI 1.85–2.93), 6.5–8% of HbA1c (OR 1.43, 95% CI 1.22–1.67), HbA1c > 8% (OR 1.96, 95% CI 1.64–2.33), 126–180 mg/dL fasting glucose (OR 1.13, 95% CI 1.01–1.26), and fasting glucose > 180 mg/dL (OR 1.90, 95% CI 1.68–2.16) had significantly higher risk of post-operative adverse events. Use of meglitinides was also associated with post-operative adverse events for patients with diabetes (OR 2.71, 95% CI 1.81–4.07).

## 4. Discussion

In this multicenter study with a propensity score-matched procedure, we found that surgical patients with diabetes had higher risks of post-operative mortality and infectious complications including septicemia, cellulitis, infectious arthritis, and acute pyelonephritis than patients without diabetes. Previous admission for diabetes, high fasting glucose, and elevated HbA1c were all significant factors associated with post-operative adverse events. Longer lengths of hospital stay and higher medical expenditure during the index surgical admission were also noted in diabetes patients.

Our results were consistent with previous studies, showing that patients with diabetes were associated with more comorbidities than those without diabetes, including hypertension, ischemic heart disease, mental disorders, cardiac dysrhythmia, heart failure, chronic renal failure, cerebrovascular disease, renal dialysis, chronic obstructive pulmonary disease, asthma, and liver cirrhosis [19,20,21]. These comorbid conditions and basic characteristics might correlate with perioperative adverse outcomes. To reduce the confounding bias, we used a propensity score-matched procedure and multivariate logistic regression to adjust the potential confounders before validating the risk of post-operative complications and mortality between patients with and without diabetes. Other potential confounding variables including age, gender, income status, types of surgery or anesthesia, duration of surgery, emergency or not, and biochemical data were also matched and adjusted in this study.

The increased risk of infection after surgeries was investigated in patients with diabetes [7,14,15,22,23]. In the present study, diabetes was significantly associated with post-operative infectious complications including septicemia, necrotizing fasciitis, cellulitis, acute pyelonephritis, and infectious arthritis. The elevation of blood glucose levels might alter immune function including polymorphonuclear phagocytosis and neutrophil chemotaxis [24,25,26], cytokines [27,28], nitric oxide-mediated microvascular relaxation [29], and immunoglobin [30,31]. Altered immune function may increase the risk of various infections and lead to failure of the surgery, delayed wound healing, sepsis, organ failure, and even death.

The impact of diabetes on post-operative adverse outcomes could be explained as follows. One explanation is that patients with diabetes have more medical conditions or more advanced comorbidities at the time of surgery; this phenomenon has been observed in previous reports and has been well adjusted for in this study. More comorbidities having an influence on perioperative outcomes is a reasonable deduction. It is necessary to consider the possibility that the metabolic abnormalities associated with diabetes are responsible for some of the increased mortality. Second, hyperglycemia intrinsically influences perioperative mortality. Hyperglycemia interferes with immune function and leads to increased risk of infectious complications [24,25,26,27,28,29,30,31]. Hyperglycemia might contribute to increased platelet activity and disordered coagulation and fibrinolytic function. Patients with hyperglycemia are especially susceptible to thrombotic events [32]. However, diabetes was not associated with increased risk of non-infectious complications in our study, and this may be because the event rate of non-infectious complications was too low to provide enough numbers for analysis.

In the present study, we found that 11.3% of surgical patients were considered to have undiagnosed diabetes (defined as people who have levels of HbA1c ≥ 6.5% or fasting glucose ≥126 mg/dL, but who have no physician’s diagnosis of diabetes). This number was reasonable according to the previous studies, showing a 5.2–17.7% prevalence of undiagnosed diabetes according to different definitions [33,34,35]. In further analysis, we found that undiagnosed diabetes was significantly associated with an increased risk of post-operative adverse events. Many studies have found that diabetes has already progressed significantly in its severity before clinical diagnosis [36,37]. During the latent phase of undiagnosed diabetes, risk factors for diabetic complications are markedly elevated and diabetic complications are developing [38]. Pre-operative surveys of fasting glucose for surgical patients should be utilized to find undiagnosed diabetes, and to provide early strategies for the prevention of post-operative adverse events in this patient group.

Hyperglycemia has been demonstrated to be a risk factor for morbidity and mortality after surgery [9,13]. We found that risk of post-operative adverse events was higher in patients with >180 mg/dL fasting glucose than in patients with 126–180 mg/dL fasting glucose. In addition, diabetic patients with an HbA1c > 8% had a higher risk of post-operative adverse events than diabetic patients with an HbA1c of 6.5–8% in this study. The study revealed that controlling fasting glucose during the pre-operative period is helpful in reducing post-operative adverse events. However, intensive insulin therapy significantly reduced morbidity, but not mortality, among all patients in the medical intensive care unit [39]. Nevertheless, we suggest that optimal glycemic control might reduce risk of post-operative adverse events in diabetic patients, but this hypothesis needs further investigation.

Some study limitations should be considered when interpreting the current results. First, the inclusion criteria of diabetic patients consisted of the physician’s diagnosis of diabetes, fasting glucose ≥ 126 mg/dL, or HbA1c ≥ 6.5% before surgical admission within 3 months. However, there were data missing before matching for diagnosis of diabetes (*n* = 9908, 56%), HbA1c (*n* = 12,586, 71%), or fasting glucose (*n* = 4214, 24%) in surgical patients, so only 1868 patients had the comprehensive data of physician’s diagnosis, fasting glucose, and HbA1c. Misclassification may have occurred, and this may contribute to underestimating the risk of post-operative adverse events in diabetic patients (bias toward the null). Second, detailed information about body mass index, lifestyles, dietary habits, and sociodemographic factors was not available in the database. The association of the potential confounders with post-operative adverse events could not be validated. Third, the wide range of CIs of several outcome risks revealed the variety and instability of our study outcome. This study limitation may be due to a small number of outcome events. Fourth, a co-linearity relationship existed between length of stay and medical expenditure in this study. Using these two events as outcomes in this study may be a study limitation because of its co-linearity. In addition, we adjusted most potential confounders, including comorbidities and biochemical data, in the match procedure and multivariate logistic regression. However, residual confounding remains possible.

## 5. Conclusions

In conclusion, diabetic patients who underwent surgery had higher risks of infectious complications and in-hospital mortality than nondiabetic patients who underwent similar major surgeries did, particularly those who had prior diabetic admissions and poor glycemic control. Routine examination of glycemic status pre-operatively is suggested to identify undiagnosed diabetes. An early intervention strategy is needed to improve the post-operative adverse outcomes in this susceptible population.

## Figures and Tables

**Table 1:** Baseline characteristics of surgical patients with and without diabetes before matching.

	No DM (*N* = 70,050)		DM (*N* = 17,660)		*p* *
Sex	*n*	(%)	*n*	(%)	<0.0001
Male	31,653	(45.2)	8658	(49.0)	
Female	38,397	(54.8)	9002	(51.0)	
Age, years					<0.0001
20–29	8726	(12.5)	366	(2.1)	
30–39	15,193	(21.7)	1277	(7.2)	
40–49	13,278	(19.0)	2430	(13.8)	
50–59	13,303	(19.0)	3903	(22.1)	
60–69	9711	(13.9)	4491	(25.4)	
70–79	5880	(8.4)	3276	(18.6)	
≥80	3959	(5.7)	1917	(10.9)	
Mean ± standard deviation	49.3 ± 17.1		60.7 ± 14.9		<0.0001
Low income	905	(1.3)	245	(1.4)	0.3193
Comorbidities					
Hypertension	6479	(9.2)	6686	(37.9)	<0.0001
Ischemic heart disease	2625	(3.7)	2610	(14.8)	<0.0001
Mental disorder	3410	(4.9)	1930	(10.9)	<0.0001
Cardiac dysrhythmias	1759	(2.5)	1126	(6.4)	<0.0001
Heart failure	953	(1.4)	1090	(6.2)	<0.0001
Chronic renal failure	578	(0.8)	1065	(6.0)	<0.0001
Cerebrovascular disease	1258	(1.8)	1071	(6.1)	<0.0001
Renal dialysis	337	(0.5)	689	(3.9)	<0.0001
COPD	859	(1.2)	511	(2.9)	<0.0001
Asthma	591	(0.8)	380	(2.2)	<0.0001
Liver cirrhosis	264	(0.4)	220	(1.2)	<0.0001
Emergency operation	7831	(11.2)	907	(5.1)	<0.0001
Types of surgery					<0.0001
Skin	696	(1.0)	206	(1.2)	
Breast	1568	(2.2)	291	(1.6)	
Musculoskeletal	14,847	(21.2)	3433	(19.4)	
Respiratory	3598	(5.1)	977	(5.5)	
Cardiovascular	954	(1.4)	605	(3.4)	
Digestive	13,944	(19.9)	3970	(22.5)	
Kidney, ureter, bladder	4047	(5.8)	1226	(6.9)	
Delivery, caesarean section	3856	(5.5)	106	(0.6)	
Neurosurgery	6035	(8.6)	2286	(12.9)	
Other	20,505	(29.3)	4560	(25.8)	
Duration of surgery, hours					<0.0001
<2	47,339	(67.6)	10,225	(57.9)	
2–4	19,684	(28.1)	5936	(33.6)	
≥5	3027	(4.3)	1499	(8.5)	
Mean ± standard deviation	1.84 ± 1.55		2.23 ± 1.99		<0.0001
Types of anesthesia					<0.0001
General	49,367	(70.5)	13,085	(74.1)	
Regional	20,683	(29.5)	4575	(25.9)	

Abbreviations: DM, diabetes mellitus; COPD, chronic obstructive pulmonary disease. * Calculated by McNemar’s test, Cochran’s Q test, or Wilcoxon signed-rank test.

**Table 2:** Baseline characteristics of surgical patients with and without diabetes after matching with propensity score.

	No DM (*N* = 16,539)		DM (*N* = 16,539)		*p*-Value *
	*n*	(%)	*n*	(%)	
Sex					0.1082
Male	8196	(49.6)	8068	(48.8)	
Female	8343	(50.4)	8471	(51.2)	
Age, years					0.4130
20–29	304	(1.8)	364	(2.2)	
30–39	1135	(6.9)	1266	(7.7)	
40–49	2369	(14.3)	2385	(14.4)	
50–59	3870	(23.4)	3754	(22.7)	
60–69	4239	(25.6)	4120	(24.9)	
70–79	2876	(17.4)	2895	(17.5)	
≥80	1746	(10.6)	1755	(10.6)	
Mean ± standard deviation	60.3 ± 14.9		60.2 ± 15.0		0.7082
Low income	213	(1.3)	226	(1.4)	0.5312
Coexisting medical conditions					
Hypertension	5515	(33.3)	5668	(34.3)	0.7629
Ischemic heart disease	1900	(11.5)	2059	(12.4)	0.2263
Mental disorder	1607	(9.7)	1638	(9.9)	0.5536
Cardiac dysrhythmias	966	(5.8)	982	(5.9)	0.6993
Cerebrovascular disease	876	(5.3)	880	(5.3)	0.9184
Heart failure	725	(4.4)	798	(4.8)	0.0964
Chronic renal failure	499	(3.0)	636	(3.8)	0.2915
COPD	449	(2.7)	455	(2.8)	0.8384
Renal dialysis	299	(1.8)	373	(2.3)	0.2134
Asthma	299	(1.8)	316	(1.9)	0.4814
Liver cirrhosis	167	(1.0)	178	(1.1)	0.5478
Emergency surgery	754	(4.6)	880	(5.3)	0.1376
Types of surgery					0.5863
Skin	182	(1.1)	189	(1.1)	
Breast	254	(1.5)	287	(1.7)	
Musculoskeletal	3245	(19.6)	3270	(19.8)	
Respiratory	923	(5.6)	920	(5.6)	
Cardiovascular	403	(2.4)	455	(2.8)	
Digestive	3759	(22.7)	3736	(22.6)	
Kidney, ureter, bladder	1171	(7.1)	1157	(7.0)	
Delivery, caesarean section	90	(0.5)	106	(0.6)	
Neurosurgery	2097	(12.7)	2087	(12.6)	
Other	4415	(26.7)	4332	(26.2)	
Duration of surgery, hours					0.9565
<2	9697	(58.6)	9687	(58.6)	
2–4	5558	(33.6)	5544	(33.5)	
≥5	1284	(7.8)	1308	(7.9)	
Mean ± standard deviation	2.19 ± 1.90		2.19 ± 1.92		0.1930
Types of anesthesia					0.4890
General	12,146	(73.4)	12,193	(73.7)	
Regional	4393	(26.6)	4346	(26.3)	

Abbreviations: DM, diabetes mellitus; COPD, chronic obstructive pulmonary disease. * Calculated by McNemar’s test, Cochran’s Q test, or Wilcoxon signed-rank test.

**Table 3:** Risks of post-operative complications and mortality outcomes of surgery patients after propensity score matching

Post-Operative Outcomes	No DM (*N* = 16,539)		DM (*N* = 16,539)		Risk of Outcomes	
	Events	Rate, %	Events	Rate, %	OR	(95%CI) *
In-hospital mortality	61	(0.4)	89	(0.5)	1.51	(1.07–2.13)
Non-infectious complications	56	(0.3)	54	(0.3)	1.01	(0.69–1.48)
Acute myocardial infarction	4	(0.0)	5	(0.0)	1.32	(0.29–5.99)
Stroke	19	(0.1)	11	(0.1)	0.60	(0.29–1.28)
Acute kidney injury	24	(0.1)	27	(0.2)	1.25	(0.71–2.20)
Post-operative bleeding	6	(0.0)	6	(0.0)	1.04	(0.33–3.28)
Pulmonary embolism	3	(0.0)	2	(0.0)	0.51	(0.08–3.39)
Deep vein thrombosis	4	(0.0)	4	(0.0)	0.94	(0.22–4.01)
Infectious complications	430	(2.6)	551	(3.3)	1.26	(1.10–1.43)
Pneumonia	104	(0.6)	88	(0.5)	0.81	(0.61–1.09)
Septicemia	96	(0.6)	132	(0.8)	1.33	(1.01–1.74)
Urinary tract infection	181	(1.1)	211	(1.3)	1.14	(0.93–1.40)
Surgical site infection	58	(0.4)	80	(0.5)	1.32	(0.94–1.86)
Fungal infection	20	(0.1)	18	(0.1)	0.88	(0.46–1.69)
Necrotizing fasciitis	3	(0.0)	15	(0.1)	3.98	(1.12–14.2)
Cellulitis	43	(0.3)	96	(0.6)	2.10	(1.46–3.03)
Acute pyelonephritis	16	(0.1)	32	(0.2)	1.86	(1.01–3.41)
Infectious arthritis	4	(0.0)	13	(0.1)	3.89	(1.19–12.7)
Osteomyelitis	9	(0.1)	16	(0.1)	1.68	(0.73–3.85)
Post-operative adverse events †	895	(5.4)	1234	(7.5)	1.45	(1.32–1.60)
Admitted to intensive care unit	1587	(9.6)	1901	(11.5)	1.24	(1.14–1.34)
Length of stay, days (mean ± SD) ‡	7.1 ± 10.1		9.1 ± 14.5		*p*-value < 0.0001	
Medical expenditure, (mean ± SD) ‡	4221 ± 4693		5167 ± 5720		*p*-value < 0.0001	

Abbreviations: OR, odds ratio; DM, diabetes mellitus; USD, United State dollars; SD, standard deviation. * Adjusted for all covariates listed in Table 2. † Post-operative adverse events included septicemia, cellulitis, acute pyelonephritis, and in-hospital mortality. ‡ In the multiple regression models, the beta coefficients of diabetes associated with length of stay and medical expenditure were 0.13 (*p* < 0.0001) and 0.19 (*p* < 0.0001), respectively.

**Table 4:** The risk of post-operative adverse events in patients with various severities of diabetes.

	*n* *	Post-Operative Adverse Events			
		Events	Rate, %	OR	(95% CI)
No diabetes	16,539	1145	(6.9)	1.00	(references)
Patients with diabetes had					
Undiagnosed diabetes	9908	757	(7.6)	1.40	(1.26–1.56)
Diabetes-related complications	2231	350	(15.7)	1.68	(1.45–1.94)
Type 1 diabetes	99	11	(11.1)	1.90	(0.96–3.75)
Types 2 diabetes	6503	736	(11.3)	1.39	(1.25–1.55)
Admission due to diabetes	725	128	(17.7)	2.33	(1.85–2.93)
HbA1c, 6.5–8%	2314	255	(11.0)	1.43	(1.22–1.67)
HbA1c, >8%	1639	194	(11.8)	1.96	(1.64–2.33)
Fasting glucose 126–180 mg/dL	8578	545	(6.4)	1.13	(1.01–1.26)
Fasting glucose >180 mg/dL	3747	457	(12.2)	1.90	(1.68–2.16)
Use of biguanides	5532	537	(9.7)	1.32	(1.03–1.70)
Use of sulfonylureas	3912	475	(12.1)	1.84	(1.39–2.42)
Use of meglitinides	1051	237	(22.5)	2.71	(1.81–4.07)
Use of dipeptidyl peptidase 4 inhibitors	2522	337	(13.4)	1.68	(1.22–2.30)
Use of α-glucosidase inhibitors	1233	178	(14.4)	1.82	(1.22–2.73
Use of glucagon-like peptide-1 agonist	325	70	(21.5)	1.98	(0.59–6.57)
Use of thiazolidinediones	571	67	(11.7)	1.43	(0.73–2.81)
Use of insulin	7797	1116	(14.3)	1.80	(1.45–2.24)

* Missing data in HbA1c (*n* = 11,175), fasting glucose (*n* = 2749), and diagnosis of diabetes (*n* = 9908).

## Supplementary Materials

The following are available online at http://www.mdpi.com/2077-0383/8/1/100/s1, Table S1: Risks of postoperative complications and mortality outcomes of surgery patients before propensity score matching, Table S2: Risks of postoperative complications and mortality outcomes of surgery patients matched sex and age.
