# Supplementary material for: Adverse Outcomes after Major Surgeries in Patients with Diabetes: A Multicenter Matched Study
Source: J Clin Med. 2019 Jan 16;8(1):100. doi: 10.3390/jcm8010100 (PMC6352271; doi:10.3390/jcm8010100)
Supplement: Supplementary file 1 [file jcm-08-00100-s001.pdf]

Table S1. Risks of postoperative complications and mortality outcomes of surgery patients before propensity score matching

	No DM (N=70050)		DM (N=17660)		Risk of outcomes
Postoperative outcomes	Events	Rate, %	Events	Rate, %	OR (95%CI)*
In-hospital mortality	126	(0.2)	105	(0.6)	1.43 (1.07-1.92)
Non-infectious complications	114	(0.2)	61	(0.3)	0.93 (0.67-1.31)
Acute myocardial infarction	6	(0.0)	5	(0.0)	1.35 (0.33-5.50)
Stroke	38	(0.1)	15	(0.1)	0.65 (0.35-1.24)
Acute kidney injury	37	(0.1)	30	(0.2)	1.16 (0.69-1.93)
Postoperative bleeding	21	(0.0)	6	(0.0)	0.96 (0.37-2.51)
Pulmonary embolism	7	(0.0)	2	(0.0)	0.71 (0.14-3.73)
Deep vein thrombosis	12	(0.0)	4	(0.0)	0.70 (0.22-2.27)
Infectious complications	1096	(1.6)	625	(3.5)	1.35 (1.21-1.51)
Pneumonia	208	(0.3)	110	(0.6)	1.01 (0.78-1.31)
Septicemia	192	(0.3)	150	(0.8)	1.48 (1.16-1.88)
Urinary tract infection	410	(0.6)	235	(1.3)	1.22 (1.02-1.45)
Surgical site infection	201	(0.3)	91	(0.5)	1.18 (0.89-1.56)
Fungal infection	31	(0.0)	20	(0.1)	1.12 (0.60-2.10)
Necrotizing fasciitis	9	(0.0)	17	(0.1)	5.42 (2.12-13.9)
Cellulitis	153	(0.2)	110	(0.6)	2.36 (1.78-3.14)
Acute pyelonephritis	48	(0.1)	34	(0.2)	2.39 (1.44-3.96)
Infectious arthritis	12	(0.0)	13	(0.1)	2.78 (1.17-6.61)
Osteomyelitis	28	(0.0)	18	(0.1)	1.47 (0.74-2.89)
Postoperative adverse events†	1933	(2.8)	1437	(8.1)	1.57 (1.45-1.70)
Admitted to intensive care unit	3774	(5.4)	2162	(12.2)	1.25 (1.16-1.34)
Length of stay, days (mean±SD) ‡	5.6±7.9		9.5±15.1		p<0.0001
Medical expenditure, (mean±SD) ‡	3128±4198		5359±5876		p<0.0001

Abbreviations: OR, odds ratio; DM, diabetes mellitus; USD, United State dollars; SD, standard deviation. *Adjusted for all covariates listed in Table 2. †Postoperative adverse events included septicemia, cellulitis, acute pyelonephritis, and in-hospital mortality. ‡The beta coefficient of diabetes associated with length of stay and medical expenditure were 0.12 (p<0.0001) and 0.18 (p<0.0001), respectively.

Table S2. Risks of postoperative complications and mortality outcomes of surgery patients matched sex and age

	No DM (N=17554)		DM (N=17554)		Risk of outcomes
Postoperative outcomes	Events	Rate, %	Events	Rate, %	OR (95%CI)*
In-hospital mortality	46	(0.3)	105	(0.6)	1.66 (1.14-2.42)
Non-infectious complications	50	(0.3)	61	(0.3)	0.86 (0.58-1.29)
Acute myocardial infarction	2	(0.0)	5	(0.0)	1.17 (0.15-9.09)
Stroke	18	(0.1)	15	(0.1)	0.55 (0.26-1.14)
Acute kidney injury	17	(0.1)	30	(0.2)	1.10 (0.58-2.08)
Postoperative bleeding	7	(0.0)	6	(0.0)	1.03 (0.32-3.25)
Pulmonary embolism	3	(0.0)	2	(0.0)	0.42 (0.06-3.05)
Deep vein thrombosis	6	(0.0)	4	(0.0)	0.72 (0.19-2.70)
Infectious complications	381	(2.2)	622	(3.5)	1.38 (1.20-1.59)
Pneumonia	91	(0.5)	109	(0.6)	0.98 (0.72-1.33)
Septicemia	81	(0.5)	150	(0.9)	1.49 (1.11-2.01)
Urinary tract infection	144	(0.8)	234	(1.3)	1.34 (1.07-1.69)
Surgical site infection	63	(0.4)	91	(0.5)	1.14 (0.80-1.62)
Fungal infection	12	(0.1)	20	(0.1)	1.11 (0.51-2.43)
Necrotizing fasciitis	4	(0.0)	17	(0.1)	3.27 (1.02-10.5)
Cellulitis	43	(0.2)	109	(0.6)	2.17 (1.49-3.16)
Acute pyelonephritis	12	(0.1)	33	(0.2)	2.49 (1.23-5.03)
Infectious arthritis	3	(0.0)	13	(0.1)	3.45 (0.89-13.5)
Osteomyelitis	13	(0.1)	18	(0.1)	0.90 (0.41-1.99)
Postoperative adverse events†	765	(4.4)	1429	(8.1)	1.62 (1.47-1.79)
Admitted to intensive care unit	1366	(7.8)	2144	(12.2)	1.27 (1.16-1.38)
Length of stay, days (mean±SD) ‡	6.6±8.9		9.5±15.1		p<0.0001
Medical expenditure, (mean±SD) ‡	3660±4109		5356±5874		p<0.0001

Abbreviations: OR, odds ratio; DM, diabetes mellitus; USD, United State dollars; SD, standard deviation. *Adjusted for all covariates listed in Table 2. †Postoperative adverse events included septicemia, cellulitis, acute pyelonephritis, and in-hospital mortality. ‡The beta coefficient of diabetes associated with length of stay and medical expenditure were 0.14 (p<0.0001) and 0.19 (p<0.0001), respectively.
